# Kinematics Adaptation and Inter-Limb Symmetry during Gait in Obese Adults

**DOI:** 10.3390/s21175980

**Published:** 2021-09-06

**Authors:** Massimiliano Pau, Paolo Capodaglio, Bruno Leban, Micaela Porta, Manuela Galli, Veronica Cimolin

**Affiliations:** 1 Department of Mechanical, Chemical and Materials Engineering, University of Cagliari, 09123 Cagliari, Italy; massimiliano.pau@dimcm.unica.it (M.P.); bruno.leban@dimcm.unica.it (B.L.); porta.micaela.ib@gmail.com (M.P.); 2 Orthopaedic Rehabilitation Unit and Clinical Lab for Gait Analysis and Posture, Istituto Auxologico Italiano IRCCS, San Giuseppe Hospital, 28824 Verbania, Italy; p.capodaglio@auxologico.it; 3 Department Surgical Sciences, Physical and Rehabilitation Medicine, University of Turin, 10124 Turin, Italy; 4 Department of Electronics, Information and Bioengineering, Politecnico di Milano, Piazza Leonardo da Vinci 32, 20133 Milano, Italy; manuela.galli@polimi.it

**Keywords:** angle-angle diagrams, cyclograms, gait, kinematics, obesity, symmetry

## Abstract

The main purpose of this study is to characterize lower limb joint kinematics during gait in obese individuals by analyzing inter-limb symmetry and angular trends of lower limb joints during walking. To this purpose, 26 obese individuals (mean age 28.5 years) and 26 normal-weight age- and sex-matched were tested using 3D gait analysis. Raw kinematic data were processed to derive joint-specific angle trends and angle-angle diagrams (synchronized cyclograms) which were characterized in terms of area, orientation and trend symmetry parameters. The results show that obese individuals exhibit a kinematic pattern which significantly differs from those of normal weight especially in the stance phase. In terms of inter-limb symmetry, higher values were found in obese individuals for all the considered parameters, even though the statistical significance was detected only in the case of trend symmetry index at ankle joint. The described alterations of gait kinematics in the obese individuals and especially the results on gait asymmetry are important, because the cyclic uneven movement repeated for hours daily can involve asymmetrical spine loading and cause lumbar pain and could be dangerous for overweight individuals.

## 1. Introduction

Obesity is a pathological condition that has a profound effect on disability and quality of life [1]. The abnormal amount of fat, which modifies the body geometry by adding passive mass to different regions, causes relevant alterations in skeletal statics and dynamics. In particular, the mass excess has been recognized to influence the biomechanics of several movements and activities of daily living, such as walking, standing up, and bending [2,3,4,5], causing functional limitations, and possibly predisposing individuals to injuries [6]. Investigating these capacities quantitatively appears necessary to define the functional profile in the obese population and then plan appropriate rehabilitation interventions.

As locomotion is one of the most important and frequent tasks in daily life, it is not surprising that the features of gait in obese individuals have been extensively investigated. Indeed, the quantification of the way obesity affects the biomechanics of gait provides important insights about the relationship between metabolic and mechanical energetics, mechanical loading (in particular at lower limb joints), and the associated risk of musculoskeletal injuries and/or pathologies. Our understanding of how obesity affects gait biomechanics is increasing, and currently a rich body of literature and several reviews [7,8,9,10] are available. However, it is noteworthy that the findings related to the effects of obesity on the kinematics and kinetics of walking are mixed. While some studies reported that obesity induces slower velocity [5,8,11,12], lower cadence [5,11,12], reduced stride length [4,5,8,12] and swing time [12,13], increased stance time [8,12,13], decreased single support time [4,8], and increased double support time [8,14], other studies failed in detecting significant changes in velocity [4,13,14,15,16], cadence [13,14,17], step length [11,14,16], stride length [17], stance time [13], single and double leg support time and swing phase duration [11]. However, the literature is consistent as regards the increased step width [4,5,14,16,17]. In addition, increased peak hip joint flexion [18], extension [12], sagittal plane range of motion (ROM) [19], and increased ankle eversion from mid-stance to pre-swing [8] have been described. Conversely, no changes in hip joint sagittal plane ROM, ankle joint peak, and ROM of eversion [18] have been found. Finally, in some cases increased hip adduction during terminal stance and pre-swing and increased knee adduction in stance and swing [8,18] have been observed as well as increased ankle plantar flexion and reduced knee flexion [12].

In summary, although the main alterations of gait pattern in obese individuals have been extensively investigated, there are some aspects which remain mostly unexplored. First, previous researches were typically conducted using discrete parameters obtained by gait analysis (angle values at specific instants of the gait cycle, range of motion, …) while no comparisons between the angle variations of hip, knee, and ankle joints in obese and normal weight individuals have been ever performed on a point-by-point basis. Comprehensive analysis of the whole angular trends during the gait cycle may provide a broader view of the gait alterations, thus representing a sound basis to plan suitable training and rehabilitative programs. Second, no data are available as regards inter-limb symmetry of obese individuals at hip, knee, and ankle joints during walking. The concept of symmetry in movement is quite controversial, as some researchers consider the human nature intrinsically asymmetrical and, as such, perfect symmetry does not exist in humans [20,21]. Nonetheless, it is commonly assumed that when a certain threshold of asymmetry is exceeded, its existence is indicative of gait alterations, which can originate from impaired motor control or from structural damage in the musculoskeletal system.

So far, different approaches have been proposed to quantify lower limb asymmetry during gait. Among discrete methods, that is those which consider single values of selected gait cycle parameters (i.e., spatio-temporal parameters or ground reaction force data [22]), the symmetry index (SI) [23,24,25,26] is one of the most commonly used. To the best of our knowledge, only one study exists about the application of SI in overweight individuals [20]; in this case, the SI was used to describe the difference between the left and right loadings considering the vertical components of the ground reaction force. It was demonstrated that a significant and high correlation is present between the SI and BMI of overweight subjects, thus suggesting that higher asymmetry of lower limb loading is associated with overweight, which implies greater risk to health of those people.

More recently, the techniques which make use of the entire angular waveforms have become widespread. In this case, inter-limb asymmetry is computed starting from continuous joint angle using bilateral cyclograms, [27,28,29,30], representing mutual dependencies between contralateral joint during the entire gait cycle [31]. Since asymmetry is usually associated with several pathologies, some studies have been conducted on this topic, in musculoskeletal, orthopaedic, and neurological diseases [27,28,32,33,34]. However, to the author’s knowledge, this approach has never been employed to obese individuals. In literature, Stodolka and Sobera [20] demonstrated that the higher postural asymmetry of the lower limb loading is associated with overweight, leading to greater risk to health of those people. Repeated asymmetry of loading both legs for hours every day can involve asymmetrical spine loading and lumbar pain [35]; this effect could be more dangerous for health in the case of overweight or obese people. A better understanding of abnormalities in gait functionality of obese individuals may result in a more detailed understanding of biomechanical factors that influence their kinematics and could give suggestions for a more appropriate and effective rehabilitation and exercise prescription. Thus, the primary goal of the present study was to investigate the existence of possible alterations in lower limb joint kinematics in obese individuals during gait using two approaches: (1) assessment of inter-limb symmetry on the basis of the angular trend of each joint calculated for the whole gait cycle and (2) assessment of the existence of possible differences, in terms of lower limb joint kinematics, with respect to normal weight individuals by means of a point-by-point comparison of the angular trends acquired during the gait cycle.

## 2. Methods

### 2.1. Participants

A convenience sample of 26 obese individuals (OW, 11 male, 15 female, mean age 28.5 years, BMI > 30 kg/m^2^, median 39.0 kg/m^2^, range 34.9–51.6 kg/m^2^) admitted for an integrated bodyweight reduction and rehabilitation program at the Istituto Auxologico Italiano, Piancavallo (VB, Italy), were recruited for the study on a voluntary basis. At the time of the experimental tests, all of them were free from any acute musculoskeletal, neuromuscular, psychological, and/or cardiopulmonary conditions able to significantly affect their walking abilities and postural control. Gait analysis data were taken from retrospective studies performed at Istituto Auxologico Italiano, Piancavallo (VB, Italy). An equal size number of normal weight individuals (BMI median 21.4 kg/m^2^, range 17.0–26.5 kg/m^2^) recruited among the hospital and University of Cagliari staff matched for age, sex, and height served as control group (NW). All participants (whose anthropometric and clinical features are reported in Table 1) were required to sign a written informed consent form, in which the details of the experimental tests were reported. The study was carried out in compliance with the World Medical Association Declaration of Helsinki and its later amendments.

### 2.2. Spatio-Temporal and Kinematic Data Collection and Processing

Spatio-temporal and kinematic parameters of gait were acquired by means of a 6-camera motion-capture system (VICON, Oxford Metrics Ltd., Oxford, UK) with a sampling rate of 100 Hz, and two force platforms (Kistler, CH). Prior to the experimental tests, the following anthropometric data were collected: height, body mass, anterior superior iliac spine distance, pelvis thickness, knee and ankle width, and leg length. Then, 22 spherical retro-reflective passive markers were placed on the individual’ skin at specific landmarks according to the protocol proposed by Davis et al. [36]. Once this preparation phase was completed, participants were requested to walk along a 10 m long walkway at their self-selected speed in the most natural manner. A trial was considered valid, and subsequently processed, if the marker trajectories were not lost during the subject’s gait and included at least one cycle per limb. At least six trials were acquired for each participant in order to guarantee reproducibility of the results. The raw 3D markers’ trajectories were thus processed using the dedicated software (Polygon Application, version 2.4, VICON, Oxford Metrics Ltd., Oxford, UK), to obtain the following variables:

- spatiotemporal parameters of gait (i.e., speed, stride length, cadence, stance, swing, and double support phase duration);
- dynamic range of motion (ROM) of hip, knee and ankle joints, calculated as difference between the minimum and the maximum angle of flexion-extension (hip and knee) and dorsi-plantarflexion (ankle) observed during the gait cycle;
- angular trends of hip, knee, and ankle joints on the sagittal plane across the gait cycle. Such curves will be subsequently used to calculate the indexes of interlimb symmetry as described later in detail.

### 2.3. Gait Symmetry Quantification by Means of Cyclograms

Synchronized bilateral cyclograms were obtained from the sagittal kinematics properly processed with a custom routine developed under Matlab environment following the procedure proposed by Goswami et al. [37]. Briefly, using right and left limb angle values acquired during the gait cycle, angle-angle diagrams for hip, knee, and ankle joints were built and, on their basis, the following symmetry parameters were extracted:

- Cyclogram area (degrees^2^): the area enclosed by the curve obtained from each angle-angle diagram [38]. In the ideal case of perfectly symmetrical gait, the cyclogram area is null, as left and right joint angles assume the same value for each time period of the gait cycle and thus all cyclogram points lie on a 45° line. Increasing values of area indicate larger interlimb asymmetry.
- Cyclogram orientation (degrees): the absolute value of the angle ϕ formed by the 45° line (i.e., perfect interlimb symmetry) and the principal axis of inertia of the cyclogram [37]. A zero value indicates perfect symmetry, while increasing values of ϕ denote higher interlimb asymmetry.
- Trend symmetry: this dimensionless parameter, calculated according to the procedure described by Crenshaw and Richards [39] is obtained by eigenvector analysis on time-normalized right and left limb gait cycles. Even in this case a null value indicates perfect symmetry, while increasing interlimb asymmetry corresponds to higher trend symmetry values.

### 2.4. Statistical Analysis

We preliminarily checked all data separately acquired for left and right limb to verify the presence of statistically significant differences between them. As they were not found, in the subsequent analysis for each participant we considered the mean value of left and right joint/limb.

The existence of differences between OW and NW groups was assessed using three different statistical approaches. In particular, one-way multivariate analysis of variance (MANOVA) was carried out to investigate the possible differences introduced by obesity in spatio-temporal parameters of gait and dynamic ROM, while one-way multivariate analysis of covariance (MANCOVA) was used in case of interlimb symmetry parameters, including gait speed as a covariate. This design allows observers to take into account in the analysis any possible differences of speed between groups which might affect, to some extent, the lower limb kinematics. In both analyses group (OW/NW) was set as the independent variable, while the dependent variables were respectively the six previously listed spatio-temporal parameters, the 3 dynamic ROM at hip, knee and ankle joints and the 3 symmetry parameters calculated for each joint. The level of significance was set at *p* = 0.05 and the effect sizes were assessed using the eta-squared (η^2^) coefficient. Univariate analysis of variance (ANOVA) was carried out as a post-hoc test by reducing the level of significance according to the Bonferroni correction.

Instead, the differences associated with the presence of obesity in joint kinematic data were assessed by comparing the “angle vs. time” curves of both groups, for each of the 3 joints of interest, on a point-by-point basis using a one-way ANOVA, using an approach previously proposed in the literature to characterize sex-related differences in kinematic patterns among population of unaffected adults (i.e., men vs. women, Bruening et al. [40]) or between healthy individuals and those affected by neurologic and orthopedic conditions [27,41,42]. In this way it was possible to define the time intervals of the gait cycle characterized by significant differences associated with obesity.

We also explored the existence of possible relationships between BMI and interlimb symmetry parameters, while controlling for gait speed, by calculating partial correlation coefficients. All statistical analyses were performed using the SPSS version 20_b_ software (IBM SPSS Statistics, Armonk, NY, USA).

## 3. Results

**Figure 1:**
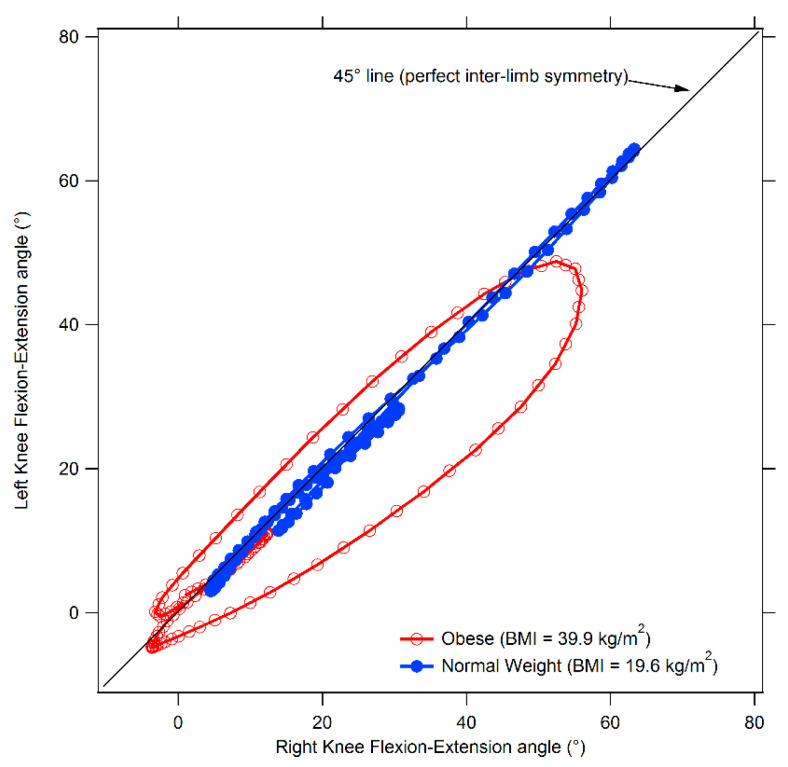
Comparison between cyclograms of obese and normal weight individuals. The diagram refers to the knee joint.

**Figure 2:**
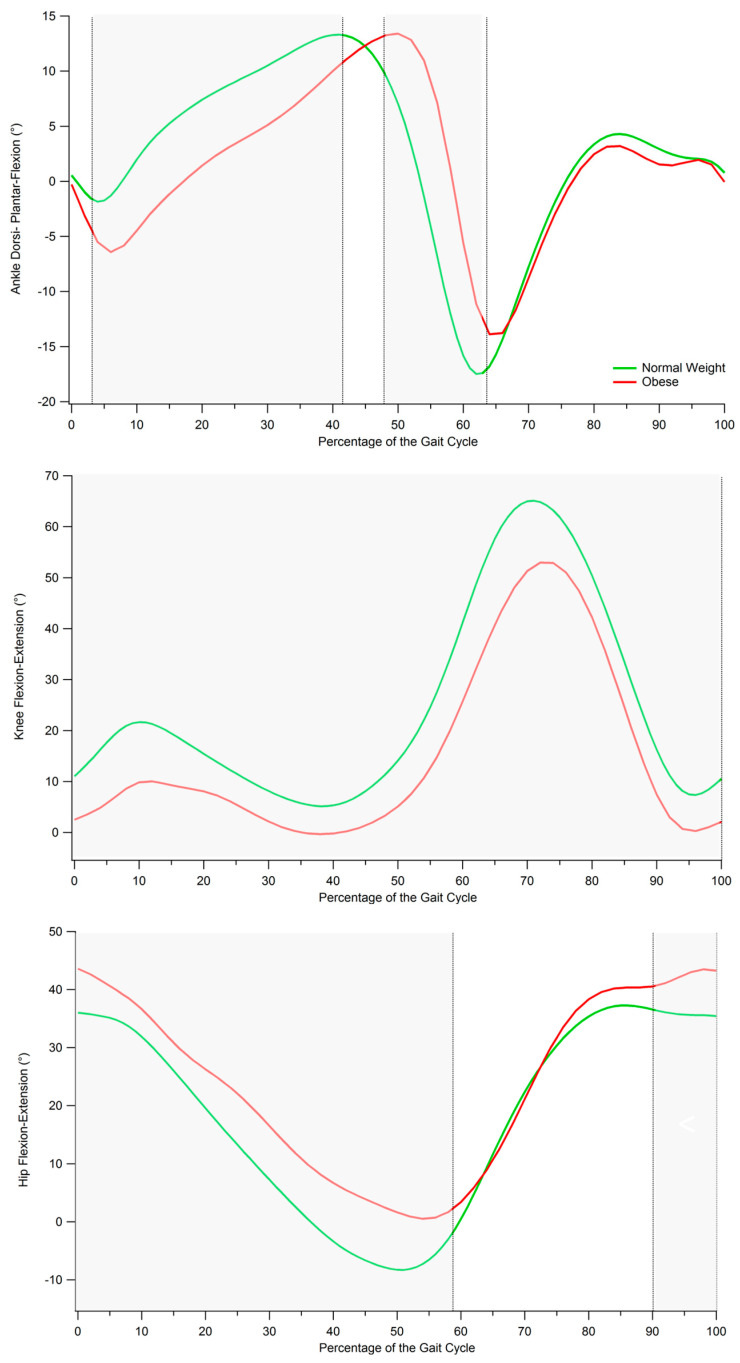
Gait kinematics in the sagittal plane for normal weight and obese individuals. From top to bottom: ankle dorsi-plantar-flexion, knee flexion-extension, and hip flexion-extension angles during gait cycle. Grey-shaded areas denote the periods of the gait cycle in which a significant difference between groups was detected (*p* < 0.05).

The results of the comparison between obese and normal weight individuals with regard to spatio-temporal gait parameters, dynamic ROM and interlimb symmetry are summarized in Table 2, Table 3 and Table 4; an example of cyclograms calculated for obese and normal weight participants are reported in Figure 1, and the angle variations in the sagittal plane during the gait cycle for hip, knee, and ankle joints are shown in Figure 2.

The statistical analysis revealed a significant effect of obesity on spatio-temporal parameters. [F(6,45) = 17.87, *p* < 0.001, Wilks λ = 0.30, η^2^ = 0.704 95% CI [0.525–0.751]]. In particular, the follow-up ANOVA showed that obese individuals were characterized by significant lower gait speed, stride length and swing phase duration and increased stance and double support phases duration.

Similarly, in case of dynamic ROM a main effect of group was detected [F(3,48) = 5.49, *p* = 0.003, Wilks λ = 0.74, η^2^ = 0.255 95% CI [0.063–0.377]]. The subsequent follow-up ANOVA revealed that obese individuals exhibited significantly reduced dynamic ankle and knee (but not hip) ROM with respect to normal weight individuals.

Finally, even in the case of symmetry parameters, after controlling for gait speed, a significant effect associated with obesity was found at knee and ankle joints (knee F(3,47) = 3.23, *p* = 0.031, Wilks λ = 0.83, η^2^ = 0.171 95% CI [0.009–0.290], ankle F(3,47) = 2.96, *p* = 0.042, Wilks λ = 0.84, η^2^ = 0.159 95% CI [0.003–0.276]). The post-hoc analysis revealed that both trend symmetry parameter for the ankle joint and cyclogram orientation at knee joint were significantly higher in the OW groups thus indicating the presence of relevant interlimb asymmetry.

The results of the correlation analysis between BMI and symmetry parameters, which are reported in Table 5, showed the existence of significant moderate positive relationship between BMI and trend symmetry at ankle joint and cyclogram orientation at knee joint.

### Point-by-Point Analysis of Kinematic Curves

The results of the analysis of hip, knee, and ankle kinematics in the sagittal plane, performed (Figure 2) on a point-by-point basis, show that significant differences between obese and normal weight individuals exist at all the three considered joints. In particular, obese individuals exhibited

- significantly increased hip flexion through all the stance phase (0 to 59% of the gait cycle) and at the end of the swing phase (90 to 100%);
- significantly reduced knee flexion through all the gait cycle;
- significantly reduced ankle dorsi-flexion for initial and mid-stance (3 to 41% of the gait cycle) and increased dorsi-flexion at the terminal stance and initial swing (48 to 64% of the gait cycle).

## 4. Discussion

The aim of the present study was to characterize the main alterations in gait kinematics in obese individuals, with special focus on interlimb asymmetry and detailed point-by-point comparison of the angular trends of hip, knee, and ankle joints between obese and normal weight individuals.

Our data indicated that most of the spatio-temporal parameters differ significantly among obese participants and controls, particularly in terms of gait speed, stride length, stance and swing phase and double support duration. Overall, such results are consistent with previous studies focused on characterizing gait patterns in obesity [4,5,8,11,12,13,14]. Taken together, the observed changes suggest the existence of a strategy specifically aimed to improve stabilization and balance control, which are threatened by the biomechanical alterations associated with mass excess. The longer duration of double support and stance phase are probably the result of a strategy aimed to allow a safer locomotion through a better optimized balanced distribution of the weight on both limbs and thus reduce the risk of instability and falls [4,43].

The kinematic pattern on the sagittal plane, as defined by the point-by-point analysis, indicates that obesity mainly affects the stance phase of gait, as most significant differences with respect to normal weight individuals were observed during that part of the gait cycle. In particular the results show that obese individuals exhibit reduced hip extension, knee flexion and ankle plantarflexion [17,44], which overall lead to a significant reduction of ankle and knee dynamic ROMs. Such alterations, which were reported (individually or in combination) in previous studies on adults and adolescents [12,45,46,47], might represent, together with reduced walking speed and longer stance phase duration, a strategy to reduce the articular stress and to compensate for the reduced muscular strength and the altered joint proprioception. Walking speed certainly plays a crucial role in defining the sagittal kinematics of gait in obese individuals and can be considered the main cause for the combination of increased knee flexion and increased plantarflexion at toe off [12]. At the same time, some researchers pointed out that walking at slower speeds may also represent the expression of a compensatory strategy aimed to limit the magnitude of the forces acting on lower extremity joints [45] and thus to reduce the risk of musculoskeletal diseases [12,17].

The significantly reduced dynamic ROMs that we detected, in particular at knee and ankle joints, may also be connected to the continuous search for stability typical of obese individuals. As they need to keep both limbs in contact with the ground, this condition increases the amount of time spent in a closed lower-limb kinematic chain condition. In this situation, the degrees of freedom of the rigid lower body system are reduced and the constraint, especially on the knee joint, increases. At last, we observe that another factor involved in the ROM reduction at knee joint might be due to the excess fat on the thigh and shank, which mechanically encumbers intersegmental rotation and counteracts the antigravity action exerted by the knee flexors [48]. As for the ankle joint, the deficits in plantar- and dorsiflexion might be due to a reduced strength of the ankle muscles, which were already reported by previous studies [49]. In this context, it is noteworthy that such effect could be reduced through suitable physical and rehabilitative intervention [50,51].

The results of the inter-limb symmetry analysis show a well-defined trend characterized by higher values of all considered parameters in obese individuals even though the statistical significance was achieved only in case of trend symmetry at ankle joint. To tour knowledge, no previous studies investigated inter-limb symmetry of lower limb joint kinematics in obese individuals, and thus there are no available data for direct comparison. However, it is noticeable that few previous studies reported the existence of significant asymmetries in spatio-temporal parameters of gait, such as step length [52] and stance phase duration [53]. Moreover, Stodolka et al. [20] calculated the symmetry index (SI) in a group of overweight individuals to quantitatively characterize the existence of possible differences between the left and right limb loading during quiet stance by analyzing the vertical components of the ground reaction force. They found that asymmetry was strongly correlated with body mass index and suggested that increased body weight may be a disadvantageous determinant of dynamic stability.

The presence of asymmetry during gait is a well-known phenomenon in neurologic conditions (i.e., multiple sclerosis, stroke [28,54] as well as in musculoskeletal disorders with a marked unilateral presentation [27]. However, in all these cases there are clear factors (e.g., either damage in a specific location of the central nervous system or injury of one limb) that justify the lack of symmetry. In case of obesity, neither of these two factors is present, and thus the reasons of the observed asymmetry should be found elsewhere. Several recent studies reported that obese individuals are characterized by uneven fat distribution in the left and right side of the body [55] and, among a group of adolescents, a larger proportion of individuals characterized by asymmetric lower limb force/power was found among obese with respect to normal weight peers [56]. At last, Bell et al. [57] suggested that lean mass asymmetries represent a co-factor in force/power asymmetry during jumping. Although we don’t have any direct evidence regarding the existence of fat/lean mass or muscular strength among left and right limb in our sample, it appears reasonable to hypothesize that asymmetry of lower limb kinematics is due to “mechanical” factors associated with differences in body composition and muscular performance of the two legs.

The described alterations of gait in our sample of obese individuals could be informative from a clinical and rehabilitative point of view. It is known that walking at a constant intensity for a prolonged time is a useful and frequently employed strategy to achieve body mass reduction in obese individuals because it is a convenient type of physical activity which involves a significant amount of metabolic energy expenditure [14]. Therefore, walking abnormalities should be carefully assessed and considered to avoid overload and possible musculoskeletal problems which would prolong the rehabilitation phase and possibly introduce negative effects. In particular, the investigation on gait asymmetry seems to be important, because the cyclic uneven movement daily repeated for hours can involve asymmetrical spine loading and cause lumbar pain [35] and this effect could be certainly more dangerous in case of individuals overweight. Even a relatively low degree of asymmetry of weight-bearing repeated every day for years could represent a co-factor for the onset of either low back pain or hip joints issues [20]. Thus, an appropriate and effective rehabilitation and exercise prescription parallel to weight loss could be tailored to recover gait pattern and reduce its asymmetry. 

This study has several limitations. Firstly, the tested sample was composed of young adults. Previous research carried out on older adults obese reported that they often exhibit articular problems (such as osteoarthritis) and severe gait alterations [58,59] that could be due to the progressive/cumulative effect of excessive joint loads over the years. Our results, which refer to young adults, could have been influenced by age-factor both in terms of gait pattern and of asymmetry which revealed a moderate severity of gait modifications with respect to controls, confirming that obesity does not determine major and immediate changes in the learned motor strategy in young adult patients. In other words, the effect of obesity on joint biomechanics seems to be not immediate, but progressive [4]. Another limitation is due to the heterogeneity of the participants in terms of severity of obesity, which makes more difficult the comparison with existing data on the literature. Finally, it is important to highlight that in this study the gait pattern was quantified using a standard marker set [36] which, in case of obese individuals, might suffer from reduced accuracy in terms of marker placement and soft tissue artefact [60,61]. Of particular concern is the marker positioning on anterior superior iliac spine (ASIS) and/or greater trochanter markers to establish the pelvis and hip joint centers. This might lead to inaccurate estimates of joint centers and, consequently, errors of the resultant kinematic/kinetic parameters, particularly as regards hip and knee [62]. However, it is also known that parameters like dynamic ROMs during gait are only slightly affected by these issues [63] and thus, even in obese, their values can be considered reliable. Future studies are needed to clearly identify the optimal marker placement as well as suitable skeletal model development procedures, to properly remove (or at very least greatly reduce) the errors possibly associated with marker placement. In this context, it is noteworthy that a combination of dual-energy X-ray absorptiometry images (to exactly assess the inter-ASIS distance and estimating segment inertial parameters [64]) with a sacral marker cluster and digitized pelvic anatomical landmarks [65] have been suggested to improve the accuracy of marker-based motion capture. Future studies should also be directed towards the investigation of specific classes of obesity to better understand its effects on gait, as the previously mentioned negative issues might be either dependent or independent by the magnitude of the mass excess.

## Figures and Tables

**Table 1:** Anthropometric and clinical features of participants. Values are expressed as mean (SD).

	Normal Weight (NW)	Obese (OW)
Participants (M, F)	26 (11M, 15F)	26 (11M, 15F)
Age (years)	28.5 (7.8)	28.7 (7.6)
Body mass (kg)	60.2 (11.9)	109.8 (15.8)
Height (cm)	165.6 (8.3)	165.5 (9.0)
Body Mass Index (kg m^−2^)	21.8 (2.8)	40.4 (0.8)

**Table 2:** Comparison between the spatio-temporal gait parameters of normal weight and obese individuals. Values are expressed as mean (SD).

	Normal Weight	Obese
Gait speed (m s^−1^)	1.30 (0.20)	1.16 (0.13) *
Stride length (m)	1.38 (0.13)	1.23 (0.10) *
Cadence (steps min^−1^)	114.54 (10.78)	112.65 (7.51)
Stance phase (% of the gait cycle)	59.15 (1.56)	61.87 (1.32) *
Swing phase (% of the gait cycle)	40.85 (1.56)	38.13 (1.35) *
Double support (% of the gait cycle)	18.44 (2.86)	47.40 (5.24) *

The symbol * denotes statistically significant difference vs. normal weight after Bonferroni correction (*p* < 0.008).

**Table 3:** Comparison between dynamic ROM of normal weight and obese individuals. Values are expressed as mean (SD).

	Normal Weight	Obese
Ankle ROM (°)	32.4 (5.7)	28.5 (5.4) *
Knee ROM (°)	61.9 (4.5)	56.0 (6.0) *
Hip ROM (°)	46.7 (5.2)	43.7 (5.3)

The symbol * denotes statistically significant difference vs. normal weight after Bonferroni correction (*p* < 0.016).

**Table 4:** Comparison between interlimb symmetry parameters of gait of normal weight and obese individuals. Values are expressed as mean (SD).

Parameter	Joint	Normal Weight	Obese
Cyclogram area (°^2^)	Ankle	77.68 (59.59)	103.84 (63.05)
Cyclogram orientation ϕ (°)		2.63 (2.04)	3.94 (4.83)
Trend Symmetry		1.34 (1.08)	2.51 (1.63) *
Cyclogram area (°^2^)	Knee	228.50 (178.97)	312.71 (221.37)
Cyclogram orientation ϕ (°)		1.09 (0.81)	2.02 (1.98) *
Trend Symmetry		0.39 (0.30)	0.70 (0.62)
Cyclogram area (°^2^)	Hip	97.22 (87.41)	124.43 (72.95)
Cyclogram orientation ϕ (°)		1.74 (1.22)	1.94 (20.6)
Trend Symmetry		0.20 (0.17)	0.68 (1.16)

The symbol * denotes a significant difference with respect to the normal weight group.

**Table 5:** Partial correlation coefficients between BMI and interlimb symmetry parameters.

Joint	Symmetry Parameter	BMI	*p*
Ankle	Cyclogram area (°^2^)	0.242	N.S.
	Cyclogram orientation ϕ (°)	0.252	N.S.
	Trend Symmetry	0.441	0.001
Knee	Cyclogram area (°^2^)	0.163	N.S.
	Cyclogram orientation ϕ (°)	0.320	0.022
	Trend Symmetry	0.245	N.S.
Hip	Cyclogram area (°^2^)	0.194	N.S.
	Cyclogram orientation ϕ (°)	−0.053	N.S.
	Trend Symmetry	0.093	N.S.

N.S. = Not Significant.

## Data Availability

Data available on request due to restrictions, e.g., privacy or ethical.
